# A nomogram prediction model for short-term aortic-related adverse events in patients with acute Stanford type B aortic intramural hematoma: development and validation

**DOI:** 10.3389/fcvm.2024.1364361

**Published:** 2024-07-10

**Authors:** Dujuan Meng, Yasong Wang, Tienan Zhou, Ruoxi Gu, Zhiqiang Zhang, Tinghao Zhao, Houlin He, Ying Min, Xiaozeng Wang

**Affiliations:** ^1^ National Key Laboratory of Frigid Zone Cardiovascular Disease, Cardiovascular Research Institute and Department of Cardiology, General Hospital of Northern Theater Command, Shenyang, China; ^2^ The General Hospital of Northern Theater Command Training Base for Graduate, Dalian Medical University, Shenyang, China

**Keywords:** type B aortic intramural hematoma, short-term exacerbation rate, nomogram, prediction model, discrimination, risk factors

## Abstract

**Background:**

This study is to examine the factors associated with short-term aortic-related adverse events in patients with acute type B aortic intramural hematoma (IMH). Additionally, we develop a risk prediction nomogram model and evaluate its accuracy.

**Methods:**

This study included 197 patients diagnosed with acute type B IMH. The patients were divided into stable group (*n* = 125) and exacerbation group (*n* = 72) based on the occurrence of aortic-related adverse events. Logistic regression and the Least Absolute Shrinkage and Selection Operator (LASSO) method for variables based on baseline assessments with significant differences in clinical and image characteristics were employed to identify independent predictors. A nomogram risk model was constructed based on these independent predictors. The nomogram model was evaluated using various methods such as the receiver operating characteristic curve, calibration curve, decision analysis curve, and clinical impact curve. Internal validation was performed using the Bootstrap method.

**Results:**

A nomogram risk prediction model was established based on four variables: absence of diabetes, anemia, maximum descending aortic diameter (MDAD), and ulcer-like projection (ULP). The model demonstrated a discriminative ability with an area under the curve (AUC) of 0.813. The calibration curve indicated a good agreement between the predicted probabilities and the actual probabilities. The Hosmer-Lemeshow goodness of fit test showed no significant difference (*χ*^2^ = 7.040, *P* = 0.532). The decision curve analysis (DCA) was employed to further confirm the clinical effectiveness of the nomogram.

**Conclusion:**

This study introduces a nomogram prediction model that integrates four important risk factors: ULP, MDAD, anemia, and absence of diabetes. The model allows for personalized prediction of patients with type B IMH.

## 1 Introduction

Aortic intramural hematoma (IMH) is a unique form of acute aortic syndrome (AAS) that differs from classic aortic dissection (AD) as it does not involve false lumen perfusion (1). According to the Stanford classification, IMH can be classified into type A and type B. Type A IMH affects the ascending aorta, while type B IMH does not involve the ascending aorta. Type B IMH accounts for 60%–70% of IMH cases (2, 3). According to the guidelines of the European Society of Cardiology(ESC) (4), initial drug therapy and repeated imaging monitoring are recommended for uncomplicated type B IMH (class I recommendation). Complicated type B IMH should be considered for thoracic endovascular aortic repair (TEVAR) (Class IIa recommendation) or surgery (Class IIb recommendation). Despite conservative drug treatment, approximately 35%–50% of patients with uncomplicated type B IMH still experience rapid disease progression (5, 6). When IMH progresses to AD or penetrating aortic ulcer (PAU), it indicates a deterioration of the natural course of IMH disease and requires high attention.

The progression and outcome of type B IMH were unpredictable during the follow-up period. Previous research has indicated that aortic-related adverse events tend to occur within the first month after the diagnosis of IMH (5, 7). However, there is a lack of studies that have developed risk prediction models to assist in clinical decision-making. Therefore, this study aims to analyze the clinical and imaging characteristics of patients with acute type B IMH and develop a simple nomogram prediction model to assess the short-term risk of deterioration in individuals with type B IMH.

## 2 Methods

### 2.1 Study population and enrollment criteria

This retrospective study was conducted at the Northern Theater General Hospital and was approved by the Ethics Committee under the ethics batch number Y(2022)151. Since this study was retrospective in nature, the Committee waived the requirement for patients’ written informed consent.

**Figure 1:**
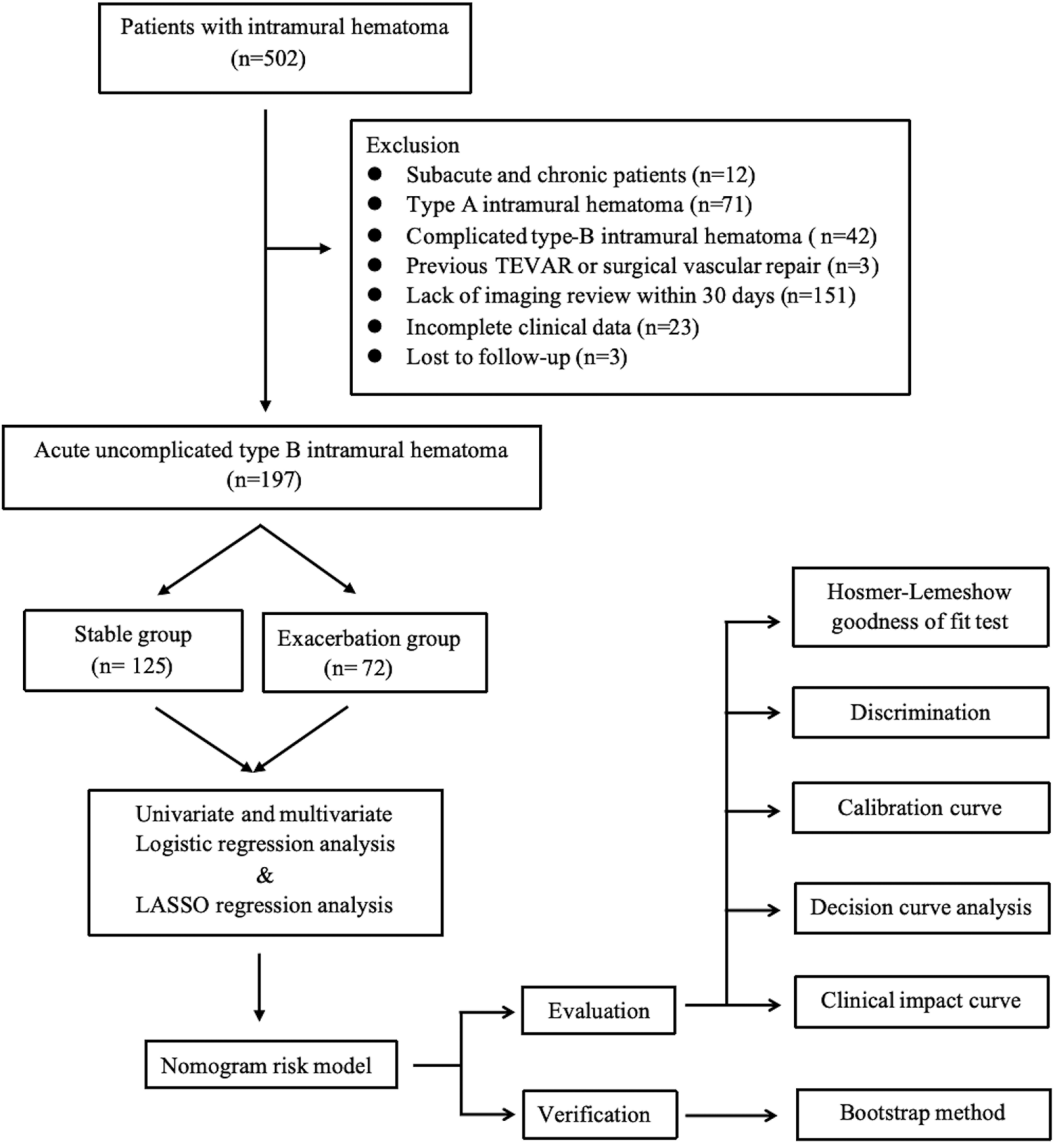
Intramural hematoma: flow chart of research. LASSO, least absolute shrinkage and selection operator; TEVAR, thoracic endovascular aortic repair.

This study conducted a continuous screening of patients diagnosed with IMH using computed tomography angiography (CTA) from July 2015 to June 2023. The screening was performed in the Emergency Department and Department of Cardiovascular Medicine of the Northern Theater General Hospital. The inclusion criteria were: (I) age ≥18 years old; (II) type B IMH. The exclusion criteria were: (I) subacute and chronic IMH with onset of more than 15 days; (II) type A IMH; (III) complicated type B IMH; (IV) previous TEVAR or surgical vascular repair; (V) traumatic aortic injury; (VI) IMH combined with Marfan syndrome and other genetic diseases and connective tissue diseases; (VII) incomplete clinical or imaging data; (VIII) lost to follow-up. A total of 197 patients with acute uncomplicated type B IMH who met the criteria were included in the study. They were divided into two groups: stable group (*n* = 125) and exacerbation group (*n* = 72). The exacerbation group is defined as patients who, within a 30-day post-discharge observation period, suffer aortic-related adverse events—such as deaths linked to aortic diseases or sudden deaths, or the progression to conditions like AD, PAU, and other complicated AAS events—despite receiving optimized medical therapy (OMT). The stable group is defined as patients who did not suffer any of the previously mentioned aortic-related adverse events during the observation period (Figure 1).

### 2.2 Treatment and management

All patients received OMT which included sedation, pain relief, blood pressure reduction, heart rate reduction, and ECG monitoring. The treatment goals were to eliminate pain and control blood pressure and heart rate. To achieve this, drugs were used to maintain blood pressure between 100 and 120 mmHg systolic and 70–80 mmHg diastolic (1 mmHg = 0.133 kPa), and heart rate between 60 and 70 beats/min. Blood pressure management primarily relied on oral anti-hypertensive drugs, with the specific medication(s) chosen based on each patient's individual situation. β-blockers were used to control heart rate, with dosage adjustments or discontinuation if the heart rate dropped below 55 beats/min.

### 2.3 CTA measurement and follow-up

All instances of IMH were diagnosed using thoracoabdominal aorta CTA with 3D reconstruction. Computed tomography was performed using a 256-detector Siemens Sensation. Intravenous administration of 80–150 ml of non-ionic contrast agent was carried out. Scans were conducted from the lower neck to the lower pelvis. All CTA images were obtained from the institutional Picture Archiving and Communication Systems. The location of the aortic lesion, aortic atherosclerosis, ulcer-like projection (ULP), hematoma shape, extent of hematoma involvement, pleural effusion, and pericardial effusion were analyzed. The maximum diameter of the ascending aorta, the maximum descending aorta diameter (MDAD), and the maximum hematoma thickness of the descending aorta (MDHT) were measured. The standard scanning protocol involved confirming the diagnosis with the initial imaging examination within 14 days of onset, followed by CTA scans at 2 weeks and 1 month. When imaging tests indicate that IMH has progressed to AD, PAU, or other high-risk conditions like impending aortic rupture, the patient may consider undergoing preventive invasive interventions to avert aortic rupture or death. The CTA images of all IMH patients were accurately diagnosed and measured by at least two experienced clinicians.

### 2.4 End points and definitions

The definition of the complicated IMH (8) encompasses the following clinical conditions: expansion of the IMH despite pharmacological management, impending rupture, uncontrolled hypertension, end-organ damage, refractory pain, or evidence of an intimal tear on CTA. Aortic-related adverse events were defined as either aortic disease-related death or sudden death, or AAS composite events such as AD and PAU. AD (9) occurs when an intrusive intimal tear divides the aorta into true and false lumens, with varying degrees of contrast medium filling both lumens. PAU (10) refers to a situation where an aortic atherosclerotic plaque penetrates the internal elastic layer into the media, creating a pocket-like protrusion on the aortic wall that is filled with contrast agent. This condition is characterized by intimal destruction and may lead to the formation of a hematoma around the protrusion. ULP (11) refers to a focal pouch filled with contrast agent that protrudes from the aortic lumen, with a breach diameter greater than 3 mm. It is different from PAU as it does not penetrate the internal elastic membrane and can occur even in the absence of atherosclerosis in patients with the disease. According to the 2014 ESC guidelines, the aorta is divided into four segments (4): the ascending aorta (from the aortic sinotubular junction to the proximal end of the brachiocephalic opening), the aortic arch (from the opening of the brachiocephalic trunk to the opening of the left subclavian artery), the descending thoracic aorta (from the distal end of the opening of the left subclavian artery to the level of the diaphragm), and the abdominal aorta (from the level of the diaphragm to the bifurcation of the iliac artery). If a hematoma involves one segment, it is recorded as 1; if two segments are involved, it is recorded as 2, and so on.

### 2.5 Statistical analysis

Statistical analysis was conducted using SPSS 26.0 and R 4.2.0. Continuous variables were presented as mean ± SD or median (quartile 1 to quartile 3). Shapiro–Wilk test and generated Quantile-Quantile plots were employed to assess whether the variables follow a normal or near-normal distribution. Independent samples *t*-test was used to compare normally distributed variables, while the Mann–Whitney *U* test was used for non-normally distributed variables. Categorical variables were reported as frequencies and percentages and compared using the *χ*^2^ or Fisher's exact test. We incorporated variables that exhibited significant differences between the two groups (*P* < 0.1) into univariate logistic regression analyses. Subsequently, the variables with *P* < 0.05 in the univariate analysis were further analyzed using multivariate analysis to further identify independent predictors. Feature selection was performed using the “glmnet” package, and factors with non-zero coefficients in the LASSO regression model were selected. The optimal penalty coefficient λ was determined using the 10-fold cross-validation method and 1 standard error (1-SE) criterion. The rms package was utilized for constructing the nomogram. To draw the receiver operating characteristic curve (ROC), calculate the area under the curve (AUC), and evaluate the model's differential diagnosis performance, the pROC package was employed. Additionally, a calibration curve was drawn to assess the calibration of the model. The rmda package was utilized for drawing decision analysis curves (DCA) and clinical impact curves (CIC) to evaluate the clinical benefit of the model. Internal validation was performed using the bootstrap method. Statistical significance was determined using a two-sided test with a *P*-value of less than 0.05.

## 3 Results

### 3.1 Baseline characteristics

This study included a total of 197 patients with type B IMH, consisting of 135 males and 62 females, with a mean age of 62.19 ± 9.79. Out of these patients, 72 (36.5%) experienced short-term aortic-related adverse events. Specifically, out of the total number of patients, 61 patients developed PAU, 8 developed AD, and 3 cases resulted in death related to aortic disease or sudden death.

In this study, the prevalence of diabetes in the type B IMH exacerbation group (1.4% vs. 12.0%, *P* = 0.009) was significantly lower compared to the stable group, while the prevalence of anemia (15.3% vs. 3.2%, *P* = 0.002) was significantly higher in the exacerbation group. These differences were statistically significant. There were no significant differences between the two groups in terms of age, gender, BMI, admission blood pressure, heart rate, drinking history, smoking history, and clinical symptoms (all *P* > 0.05). Please refer to Tables 1, 2.

**Table 1:** Clinical characteristics.

Variable	Stable group (*n* = 125)	Exacerbation group (*n* = 72)	*P* value
Age, year	63.10 ± 10.08	61.64 ± 9.30	0.314
Male, *n* (%)	81 (64.8)	54 (75.0)	0.138
BMI, kg/m^2^	25.14 ± 3.62	24.77 ± 3.47	0.489
Heart rate, BPM	78 (70,90)	81.5 (75,89.5)	0.085
Systolic BP, mmHg	138 (124,155)	139 (127,150)	0.915
Diastolic BP, mmHg	82 (75,95)	82 (76,90)	0.389
Drinking, *n* (%)	58 (46.4)	39 (54.2)	0.294
Smoking, *n* (%)	72 (57.6)	43 (59.7)	0.771
Hypertension, *n* (%)	88 (70.4)	51 (70.8)	0.949
Diabetes, *n* (%)	15 (12.0)	1 (1.4)	0.009
Coronary artery disease, *n* (%)	6 (4.8)	4 (5.6)	1.000
Cerebrovascular disease, *n* (%)	10 (8.0)	8 (11.1)	0.466
Anaemia^a^, *n* (%)	4 (3.2)	11 (15.3)	0.002
Renal cyst, *n* (%)	27 (21.6)	18(25.0)	0.584

Data presented as as mean ± SD, median (quartile 1 to quartile 3) or number (percentage). BMI, body mass index; BPM, beat per minute; BP, blood pressure.

^a^ Anemia is defined as a hemoglobin level of <120 g/L in adult males and <110 g/L in adult females.

**Table 2:** Clinical symptoms.

Variable	Stable group (*n* = 125)	Exacerbation group (*n* = 72)	*P* value
Chest or back pain, *n* (%)	119 (95.2)	68 (94.4)	1.000
Abdominal pain, *n* (%)	12 (9.6)	5 (6.9)	0.523
Low back pain, *n* (%)	3 (2.4)	1 (1.4)	1.000
Pain of radiation, *n* (%)	7 (5.6)	2 (2.8)	0.576
chest distress, *n* (%)	17 (13.6)	9 (12.5)	0.826
dyspnea, *n* (%)	24 (19.2)	15 (20.8)	0.782

Data presented as number (percentage).

Laboratory examination results revealed that the type B IMH exacerbation group had significantly lower levels of glucose [6.14 (5.22, 7.41) mmol/L vs. 7.08 (6.03, 8.66) mmol/L, *P* < 0.001] compared to the stable group. On the other hand, the type B IMH exacerbation group had significantly higher levels of creatinine [68.53 (57.35, 87.60) µmol/L vs. 65.60 (49.88, 79.70) µmol/L, *P* = 0.034], C-reactive protein [11.80 (2.48, 48.90) mg/L vs. 6.15 (2.00, 14.23) mg/L, *P* = 0.043], and D-dimer level [2.31 (1.01, 4.38) mg/L vs. 1.30 (0.63, 2.87) mg/L, *P* = 0.008] compared to the stable group. These differences were statistically significant. However, there were no significant statistical differences in white blood cell count, platelet count, serum low-density lipoprotein C, creatine phosphokinase-myocardial band, troponin T, alanine aminotransferase, and aspartate aminotransferase between the two groups (all *P* > 0.05) (Table 3).

**Table 3:** Laboratory results.

Variable	Stable group (*n* = 125)	Exacerbation group (*n* = 72)	*P* value
WBC, 10^9^ /L	11.10 ± 3.12	10.50 ± 3.19	0.147
HB, g/L	139.06 ± 15.06	133.88 ± 18.76	0.035
PLT, 10^9^ /L	217.70 ± 82.04	220.93 ± 66.18	0.408
LDL-C, mmol/L	2.94 ± 1.09	2.79 ± 0.69	0.511
CK-MB, U/L	12.00 (9.00,15.00)	11.10 (8.80,14.28)	0.318
TNT, μg/L	0.10 (0.01,8.50)	3.00 (0.01,9.00)	0.439
ALT, U/L	18.02 (13.08,25.60)	16.57 (13.10,24.36)	0.696
AST, U/L	20.21 (17.00,23.70)	20.41 (16.33,24.53)	0.842
CR, µmol/L	65.60 (49.88,79.70)	68.53 (57.35,87.60)	0.034
CRP, mg/L	6.15 (2.00,14.23)	11.80 (2.48,48.90)	0.043
D-dimer, mg/L	1.30 (0.63,2.87)	2.31 (1.01,4.38)	0.008
GLU, mmol/L	7.08 (6.03,8.66)	6.14 (5.22,7.41)	<0.001

Data presented as mean ± SD, or median (quartile 1 to quartile 3). WBC, white blood cell; HB, hemoglobin; PLT, platelet count; LDL-C, serum low-density lipoprotein C; CK-MB, creatine phosphokinase-myocardial band; TNT, troponin-T; ALT, alanine aminotransferase; AST, aspartate aminotransferase; CR, creatinine; CRP, C-reactive protein; GLU, glucose.

The results of the imaging examination revealed that the proportion of MDAD (37.97 ± 4.64 mm vs. 35.09 ± 4.76 mm, *P* < 0.001), MDHT (9.77 ± 3.27 mm vs. 8.83 ± 3.16 mm, *P* = 0.049), and ULP (41.7% vs. 9.6%, *P* < 0.001) were significantly higher in the exacerbation group compared to the stable group. These differences were statistically significant (all *P* < 0.05). However, there were no significant statistical differences between the two groups in terms of pleural effusion, pericardial effusion, maximum ascending aorta diameter, aortic atherosclerosis, number of hematoma-involved segments, IMH passing diaphragm, IMH passing iliac artery, crescent hematoma, and circular hematoma (all *P* > 0.05) (Table 4).

**Table 4:** CTA characteristics.

Variable	Stable group (*n* = 125)	Exacerbation group (*n* = 72)	*P* value
Pericardial effusion, *n* (%)	47 (37.6)	30 (41.7)	0.573
Pleural effusion, *n* (%)	7 (5.6)	9 (12.5)	0.088
Maximum ascending aorta diameter, mm	42.65 ± 4.93	43.39 ± 4.15	0.281
MDAD, mm	35.09 ± 4.76	37.97 ± 4.64	<0.001
MDHT, mm	8.83 ± 3.16	9.77 ± 3.27	0.049
Aortic atherosclerosis, *n* (%)	44 (35.2)	33 (45.8)	0.141
ULP, *n* (%)	12 (9.6)	30 (41.7)	<0.001
Segment numbers involved	2.38 ± 0.63	2.40 ± 0.57	0.902
1 segment	10 (8.0)	3 (4.2)	0.456
2 segment	57 (45.6)	37 (51.4)	0.433
3 segment	58 (46.4)	32 (44.4)	0.791
IMH passing diaphragm, *n* (%)	106 (84.8)	66 (91.7)	0.163
IMH passing iliac artery, *n* (%)	16 (12.8)	13 (18.1)	0.316
crescent hematoma, *n* (%)	73 (58.4)	41 (56.9)	0.842
circular hematoma, *n* (%)	52 (41.6)	31(43.1)	0.842

Data are expressed as mean ± SD, or number (percentage). CTA, computed tomography angiography; MDAD, maximum descending aorta diameter; MDHT, maximum descending aorta hematoma thickness; ULP, ulcer-like projection; IMH, intramural hematoma.

### 3.2 Logistic regression analysis and LASSO regression analysis

**Figure 2:**
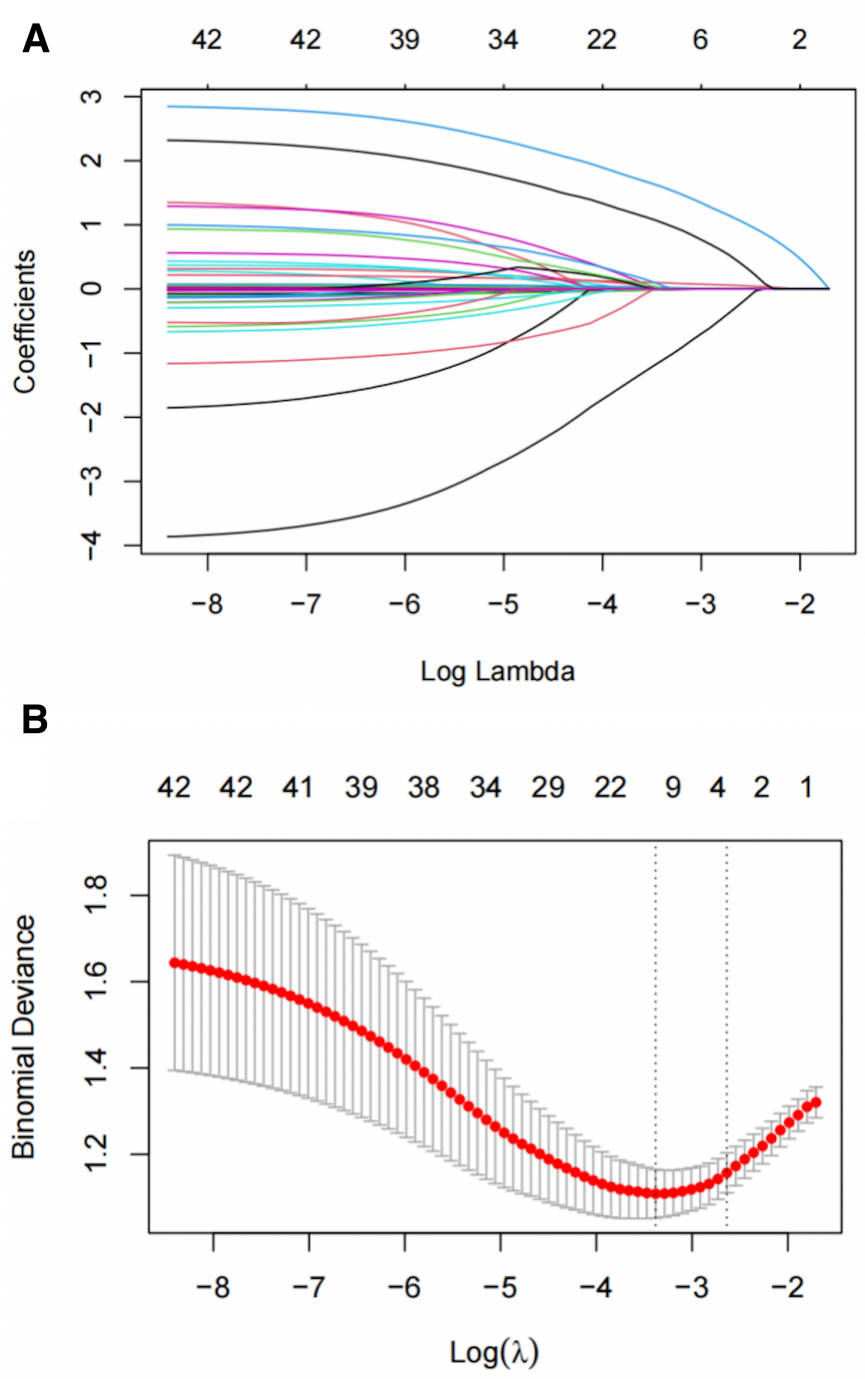
Short-term deterioration predictors of patients with type B IMH screened by LASSO regression. (**A**) LASSO coefficient distribution map for each clinical feature. (**B**) 10-fold cross-validation and 1-SE criteria determine the best penalty coefficient λ for LASSO models. SE, standard error; LASSO, least absolute shrinkage and selection operator.

Logistic regression analysis was conducted to examine the relationship between clinical data, imaging characteristics, and the occurrence of aortic-related adverse events in type B IMH. The results revealed that diabetes (OR = 0.053, 95%CI: 0.005–0.580, *P* = 0.016), anemia (OR = 6.814, 95%CI: 1.761–26.364, *P* = 0.005), MDAD (OR = 1.149, 95%CI: 1.066–1.238, *P* < 0.001), ULP (OR = 8.941, 95%CI: 3.708–21.561, *P* < 0.001) were independent predictors of aortic-related adverse events. These predictors are summarized in Table 5. To further validate the predictors, LASSO regression analysis was performed, which confirmed the same four predictors as the logistic regression analysis, thereby strengthening our model (Figure 2).

**Table 5:** Logistic regression analysis of the risk factors of intramural hematoma deterioration.

Variable	Univariate analysis		Multivariate analysis	
	OR (95% CI)	*P* value	OR (95% CI)	*P* value
Heart rate	1.019 (0.998–1.039)	0.070		
Diabetes	0.103 (0.013–0.799)	0.030	0.053 (0.005–0.580)	0.016
Anaemia	5.455 (1.668–17.842)	0.005	6.814 (1.761–26.364)	0.005
CR	1.006 (0.998–1.013)	0.142		
CRP	1.007 (1.000–1.014)	0.054		
D-dimer	1.053 (1.001–1.109)	0.048	1.060 (0.998–1.125)	0.057
Pleural effusion	2.408 (0.856–6.772)	0.096		
MDAD	1.136 (1.064–1.213)	<0.001	1.149 (1.066–1.238)	<0.001
MDHT	1.094 (0.999–1.198)	0.053		
ULP	6.726 (3.154–14.345)	<0.001	8.941 (3.708–21.561)	<0.001

Data presented as adjusted odds ratio (OR) with a 95% confidence interval (CI). CR, creatinine; CRP, C-reactive protein; MDAD, maximum descending aorta diameter; MDHT, maximum descending aorta hematoma thickness; ULP, ulcer-like projection.

### 3.3 Establishment and application of nomogram model

**Figure 3:**
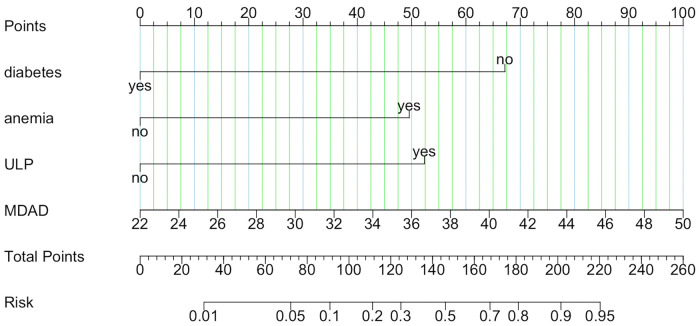
A nomogram risk model predicting short-term deterioration in patients with type B IMH.

**Figure 4:**
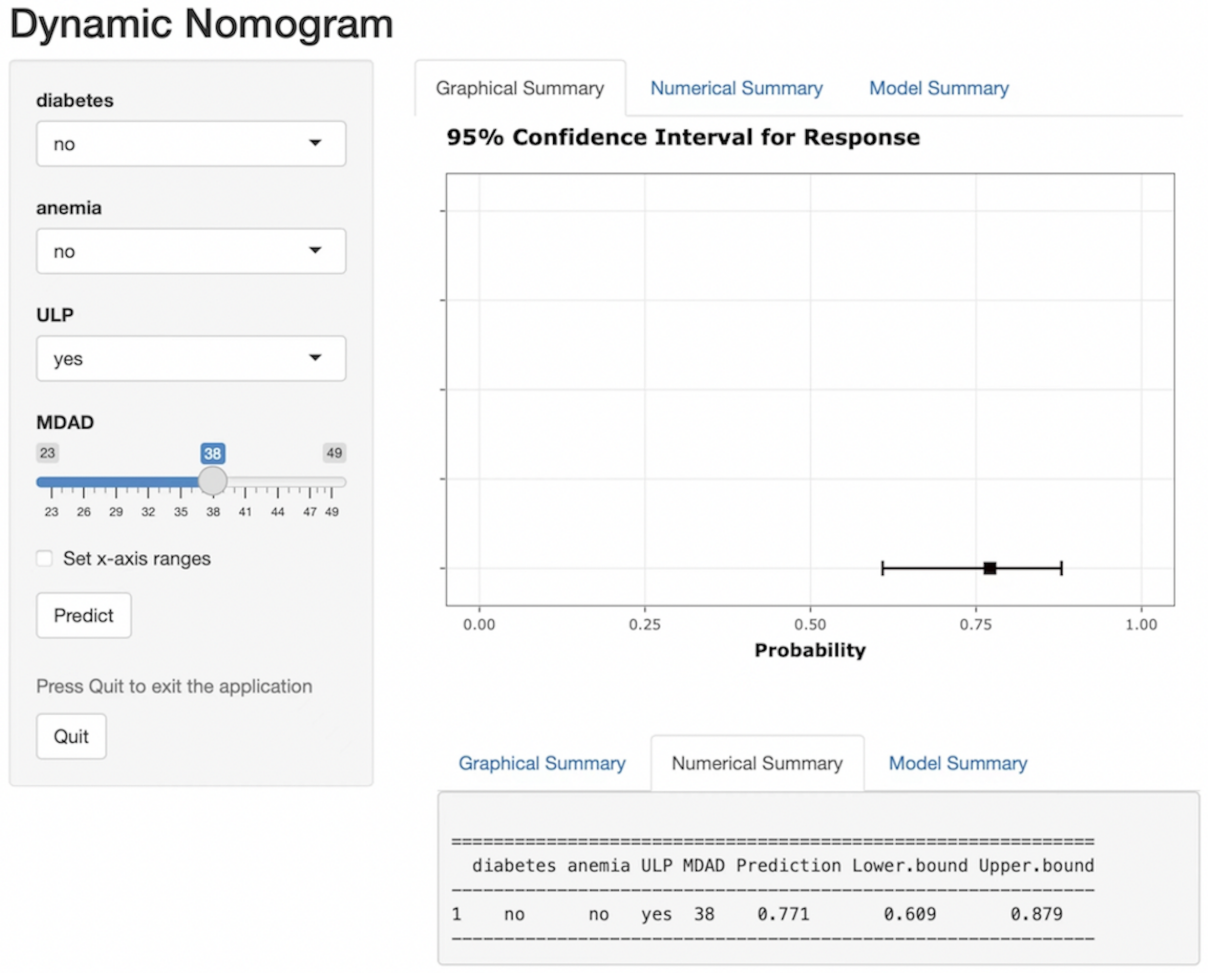
Web-based dynamic nomogram.

A nomogram risk prediction model for short-term exacerbation in type B IMH patients was established based on the 4 statistically significant influencing factors obtained from Logistic regression and LASSO regression analysis. Each risk factor was evaluated to calculate the predicted probability of short-term exacerbation risk for patients with type B IMH. Figure 3 illustrates that each variable corresponds to a line segment, with the scale indicating the value. The total score is calculated by adding the corresponding scores above each variable, and based on this total score, the predicted probability of the patient's recent deterioration risk is obtained. To address the limitations of nomograms in practical applications, this study developed a web-based nomogram dynamic prediction tool (Figure 4) (https://imh2023.shinyapps.io/DynNomapp/). This tool facilitates easy calculation and assists in clinical decision-making. As an example, if a patient has no history of diabetes or anemia, and exhibits imaging manifestations accompanied by ULP, along with an MDAD of 38 mm, the results indicate a predicted risk of aortic-related adverse events in the patient to be 77.1%. This suggests a high risk of recent deterioration for the patient.

### 3.4 Validation and efficiency evaluation of nomogram model

**Figure 5:**
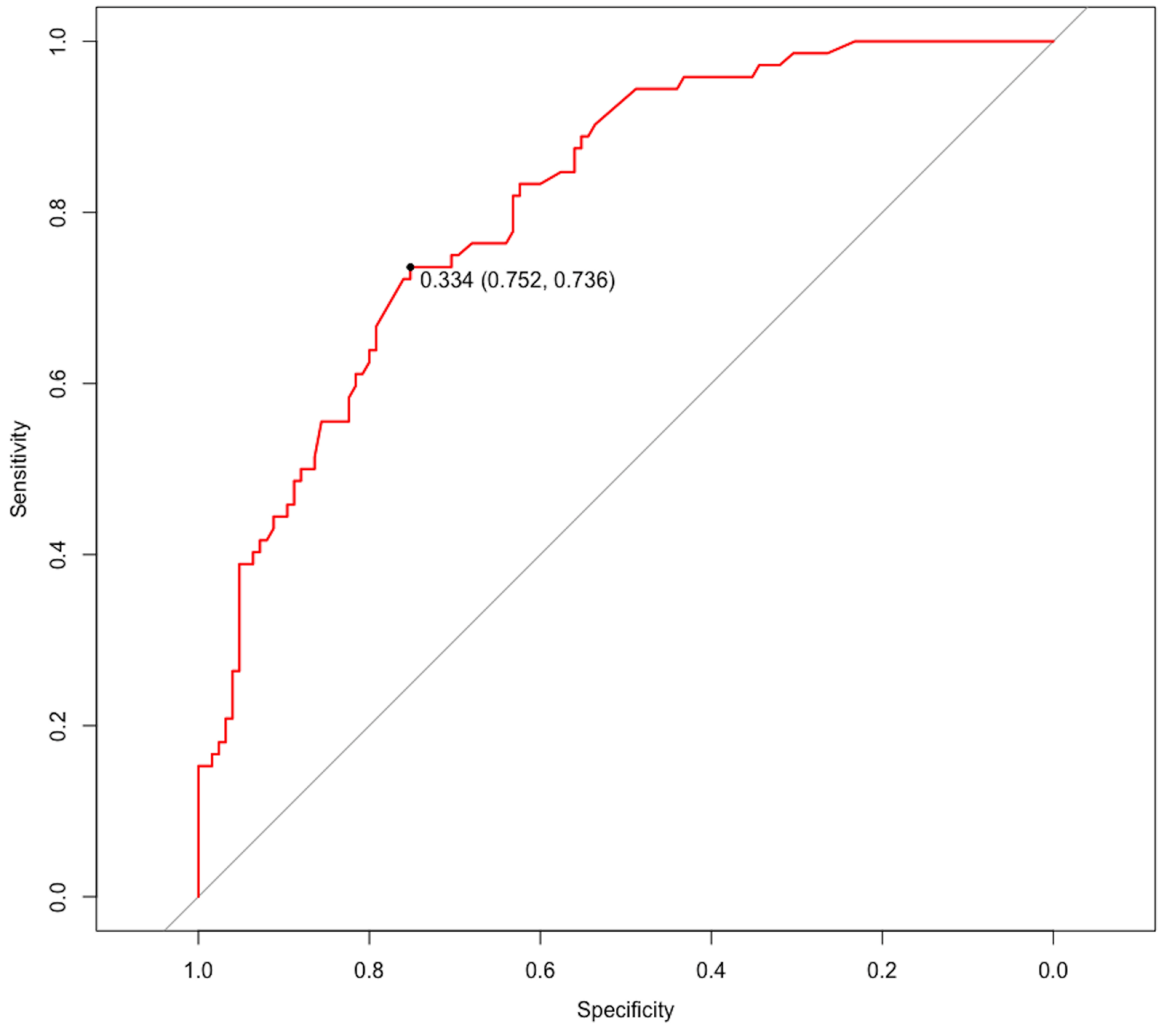
ROC curve for evaluating the model's discrimination performance. ROC, receiver operating characteristic.

**Figure 6:**
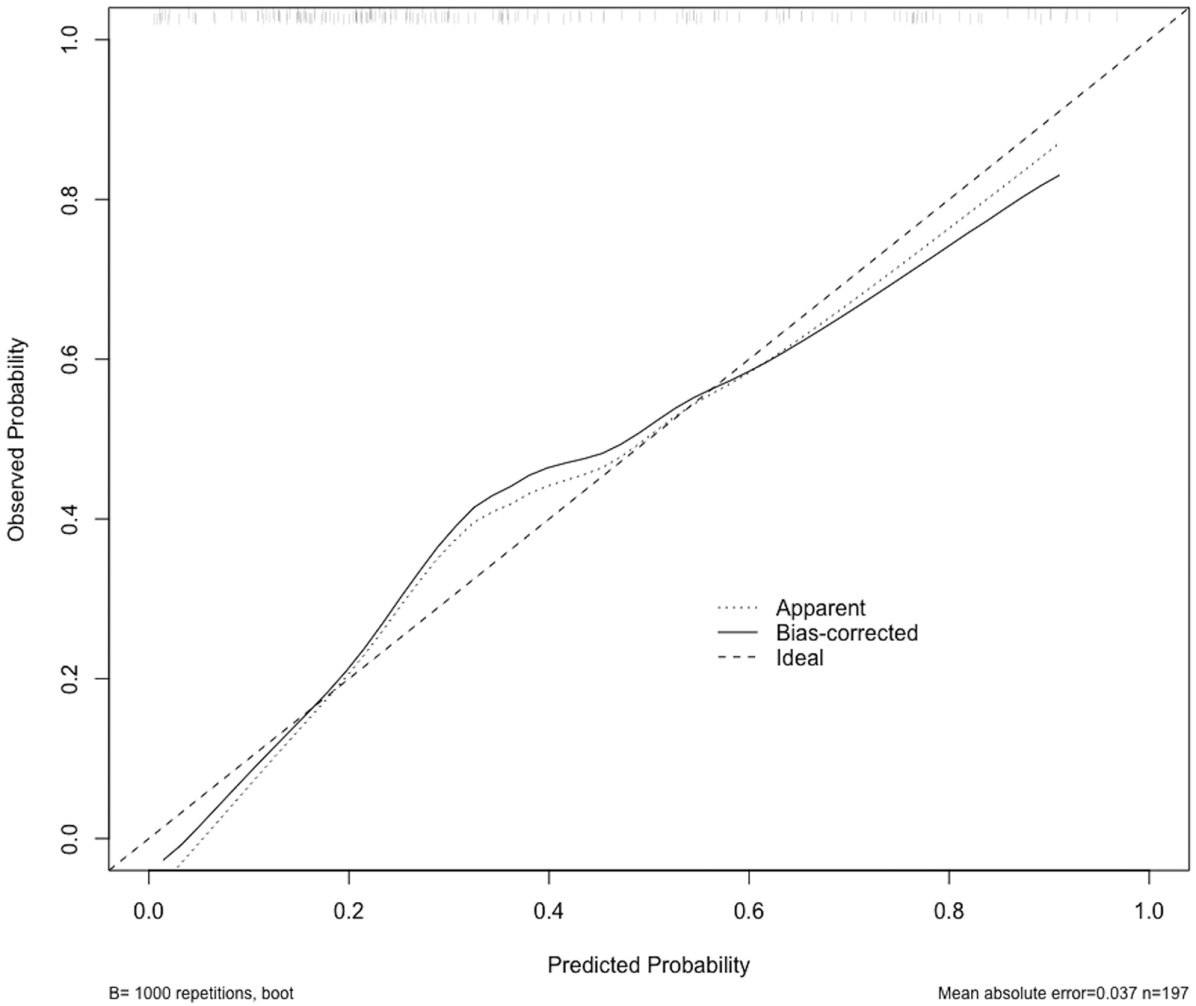
The calibration curve shows the relationship between the predicted probability of occurrence (x-axis) and the actual probability of occurrence (y-axis). The “Ideal” line represents the ideal prediction model. The “Apparent” line represents the prediction probability of this model in the original development data set. The solid black line represents the prediction probability obtained through Bootstrap resampling, which involved resampling the original development data set 1,000 times.

The ROC curve was generated based on the nomogram risk model, and the AUC was 0.813 (95%CI, 0.756–0.872), indicating a strong predictive performance of the model. At a Youden index of 0.334, the model demonstrated a specificity of 0.752 and a sensitivity of 0.736 (Figure 5). To validate the model, the Bootstrap method was employed with 1000 repetitions, resulting in an AUC of 0.794. The calibration curve demonstrated good agreement between the predicted and actual probabilities of the type B IMH short-term exacerbation risk model (Figure 6), with an accuracy rate of 0.724, indicating the model’s reliability. Furthermore, the Hosmer–Lemeshow goodness-of-fit test yielded a non-significant result (*χ*^2^ = 7.040, *P* = 0.532), confirming the model's stability.

### 3.5 Clinical application of nomogram model

**Figure 7:**
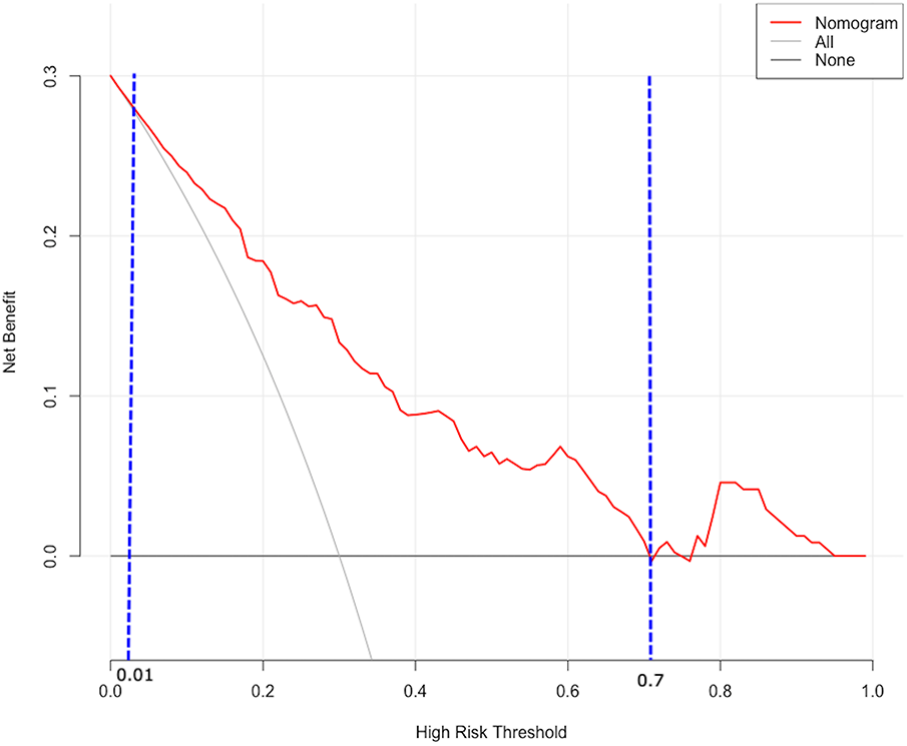
DCA for predictive nomogram. The X-axis represents the threshold, while the Y-axis represents the net benefit. The “None” line represents the net benefit of interventions without involving patients. The “All” line represents the net benefit of interventions involving all patients. The solid red line represents the net benefit of interventions specifically for patients. DCA, decision curve analysis.

**Figure 8:**
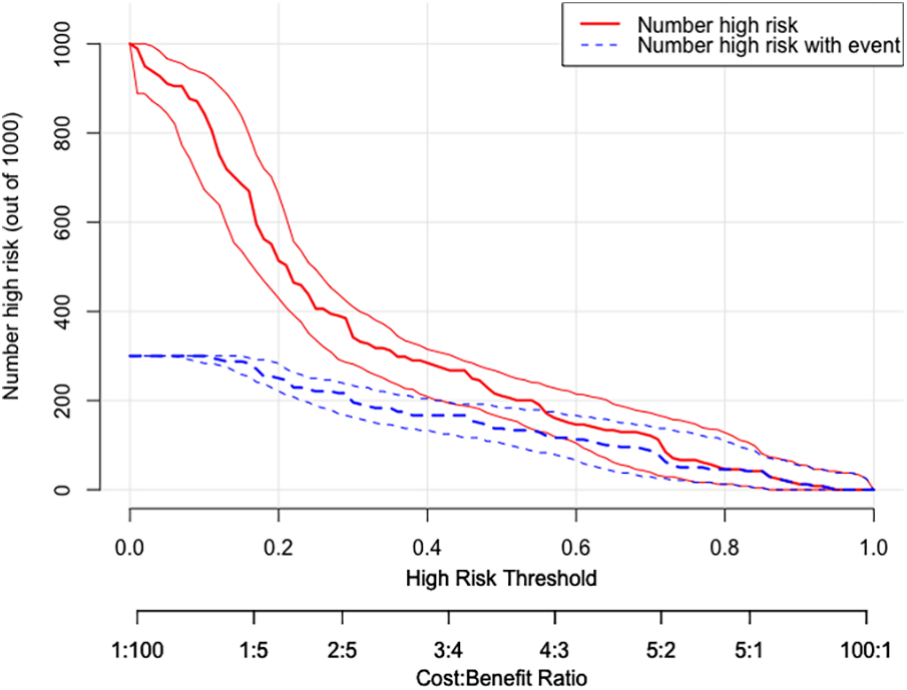
CIC of the nomogram. The two horizontal axes depict this correspondence. The solid red lines indicate the total number of high-risk patients for each risk threshold out of a sample size of 1,000 patients. The dashed blue line represents the number of actual positive events that occur in high-risk patients. CIC, clinical impact curves.

DCA was utilized to assess the probability of short-term exacerbation in patients with type B IMH, aiming to determine whether the model yielded a significant net benefit. According to this study, DCA revealed that our predictive model generated more clinical net benefit when the risk threshold was set within the range of 0.01–0.7, compared to the options of “no intervention” or “all intervention” (Figure 7). Additionally, CIC plots were employed to illustrate the number of high-risk patients and the number of truly high-risk cases at each high-risk threshold, thereby demonstrating the model's favorable predictability and clinical applicability (Figure 8).

## 4 Discussion

This study is a retrospective analysis of a specific patient population in China, focusing on acute uncomplicated type B IMH. The study aimed to identify independent predictors of short-term aortic-related adverse events in these patients by evaluating clinical, laboratory, and imaging data from 197 patients with type B IMH. Our study found that ULP, MDAD, anemia, and absence of diabetes were identified as independent predictors. Additionally, the study aimed to develop an easy-to-use nomogram for predicting the risk of short-term deterioration based on these independent predictors. The nomogram model was evaluated and internally validated, and it was found to be effective in predicting the short-term prognosis of type B IMH patients treated with OMT.

The natural history of disease in type B IMH is uncertain, with a range of outcomes including spontaneous regression of hematoma, progression to PAU, AD, aneurysm, and even aortic rupture (12, 13). In our study, out of 197 patients, 72 (36.5%) developed AD, PAU, or aortic disease-related death or sudden death within 30 days of admission. Among these patients, 39 underwent TEVAR, 2 underwent surgical vascular replacement, and 28 opted for conservative treatment with drugs. In the patients who underwent TEVAR, one patient died of respiratory failure five months after surgery. Additionally, two patients experienced cerebral infarction, one occurrence was observed one year after TEVAR, and the other occurred two years after TEVAR. Postoperative reexamination images were obtained from 15 patients, revealing that two patients developed partial ulcers at the distal end of the stent at one month and four months after the operation. Furthermore, one patient developed a small amount of new endoleak two years after the operation. The remaining patients who underwent reexamination exhibited proper adherence of the stent to the vessel wall. Among the patients who declined surgical treatment, one patient died of bone metastasis from lung cancer 3.5 years after being diagnosed with IMH. Another patient experienced two episodes of cerebral hemorrhage, one and two years after the diagnosis of IMH, and is currently in a coma. Follow-up images were obtained for eight patients, out of which two had stable ulcers, four had enlarged ulcers, one patient underwent TEVAR treatment after being admitted to the hospital, and the remaining three patients still refused surgery. Additionally, two patients had worsened AD compared to before. Due to the high surgical risk, they opted to continue conservative drug treatment. In the stable group, a total of 17 patients underwent repeated CTA measurements. Out of these, 11 patients had their hematoma completely absorbed, 2 patients developed into PAU, 1 patient was hospitalized for TEVAR, 1 patient declined surgical treatment, 1 patient had thoracic aortic dilatation, and 1 patient had aneurysm-like dilation of the aortic arch. Additionally, there was 1 new case of renal aneurysm. Therefore, we believe that close medical monitoring and timely surgical intervention within the first month are crucial for improving the prognosis of patients with type B IMH.

IMH is a highly unstable disease with an unknown pathogenesis. It is commonly believed to occur due to rupture and bleeding of the vasa vasorum within the aortic wall. The hematoma is typically found outside the media adjacent to the adventitia, without any intimal tears, and it does not directly communicate with the aortic lumen. As a result, the classic intimal flap seen in aortic dissection does not form (8, 14). In the past, early CTA examinations were limited by the spatial and temporal resolution of the equipment, making it difficult to detect small intimal tears. However, with the advancement of imaging technology, the identification rate of intimal tears in CTA scans of IMH patients has increased. More and more “small tears” have been confirmed to exist in IMH (15–18). Therefore, some scholars propose that IMH may be caused by tiny tears in the aortic intima, leading to a flow communication between the true and false lumen (11, 15, 19). Of the 38 IMH patients reported by Kitai et al. (15), 23.7% had intimal defects. In the study by Wei et al. (16), ULP was found in 23.81% of IMH patients during the initial CTA examination. In our study, 42 patients had ULP detected during the first CTA scan, with a detection rate of 21.3%, which is consistent with previous findings. Furthermore, several studies have demonstrated the negative impact of ULP on the prognosis of IMH (4, 13, 20). In our study, the exacerbation group with ULP accounted for 41.7%, significantly higher than the 9.6% in the stable group. Type B IMH patients with acute ULP had a higher risk of short-term exacerbation, indicating a poor prognosis. These findings suggest that small intimal tears play a significant role in the development of IMH. Therefore, IMH patients with ULP should undergo regular imaging follow-up and receive active treatment.

Previous studies have demonstrated that MDAD is a risk factor for poor prognosis in patients with IMH (18, 21). This study revealed that the MDAD of patients in the exacerbation group was higher compared to the stable group. As the MDAD increased, the likelihood of aortic-related adverse events in IMH patients also increased. The baseline MDAD (OR = 1.149, 95%CI: 1.066–1.238, *P* < 0.001) independently predicted aortic-related adverse events. Furthermore, other studies have indicated (22) that an increase in MDHT is associated with the progression of IMH. In our study, the baseline MDHT of the deterioration group was higher than that of the stable group, and this difference was statistically significant. However, logistic regression analysis demonstrated that MDHT was not a predictor of aortic-related adverse events, which aligns with the findings of Li et al.'s research (5). This finding supports the rationale behind our results. Given the limited sample size of our study, further large-scale, multi-center research is necessary to validate these findings.

Park et al. (18) reported on a study of 107 patients, among whom 30 (28.0%) experienced progression of IMH. These patients had significantly lower initial hemoglobin concentrations. In our study, we defined anemia as a hemoglobin level below 120 g/L for men and 110 g/L for women, and found that it was an independent risk factor for short-term aortic adverse events in patients with type B IMH. The relationship between IMH and anemia can be explained as follows: First, anemia reduces the oxygen-carrying capacity of cells and leads to insufficient tissue oxygen supply, which in turn affects aortic function. Additionally, studies have shown (23) that blood shear stress can promote the growth and differentiation of vascular endothelial cells, enhance blood vessel stability, and prevent vessel rupture and bleeding. Hypoxic supply can also lead to sympathetic nerve excitement, resulting in increased blood pressure, heart rate, ventricular contractility, aortic blood flow velocity, and blood shear force, ultimately leading to the progression of arterial disease in the aorta.

Previous studies (24) have indicated a low incidence of diabetes in patients with IMH, suggesting a negative correlation between diabetes and IMH incidence. Furthermore, hyperglycemia has been found to potentially play a protective role in the development of IMH. Similarly, the protective value of diabetes in AD and aortic aneurysm has been confirmed (25–27). A study by Chen et al. (28) observed that in the acute phase, the non-diabetic group had a disease progression incidence of 22%, whereas the diabetic group had only 7%. The average progression time of the disease was significantly shorter in the non-diabetic group (6.4 days) compared to the diabetic group (12 days), highlighting a better prognosis for type B IMH patients with diabetes. Our study also supports the conclusion that diabetes acts as a protective factor against aortic-related adverse events in type B IMH. However, the mechanism by which diabetes prevents the development of aortic disease is still not fully understood. There are currently multiple mechanisms proposed for the relationship between diabetes and aortic disease. One mechanism suggests that matrix metalloproteinases (MMPs) play a role in regulating the formation of aortic aneurysms and promoting the rupture of atherosclerotic plaques (29). It has been observed that the secretion of MMPs from aortic inflammatory cells is reduced in diabetic patients, and high blood sugar levels can inhibit the activation of MMPs in the bloodstream, leading to a decrease in the degradation of the aortic wall (30). Another mechanism suggests that diabetes can directly affect the progression of aortic disease through metabolic effects and modulation of inflammation in the aortic wall. Hyperglycemia has been found to decrease the infiltration of inflammatory cells into the aortic media and inhibit the formation of new blood vessels in the aortic adventitia. This, in turn, reduces the death of vascular smooth muscle cells and the degradation of the extracellular matrix, ultimately inhibiting the progression of aortic disease (31).

The current study has several limitations to consider. It is important to note that this study is a single-center retrospective study, which could introduce selection bias during the case selection process. Additionally, the sample size was restricted due to the low incidence of IMH in the general population. While external validation from other centers was not obtained, internal validation through bootstrapping indicated good calibration and discrimination of the model. Furthermore, a portion of patients were excluded from the study as they were referred back to a different center for further follow-up, resulting in a lack of timely imaging and clinical data for these individuals.

## 5 Conclusion

In this study, we analyzed the clinical data and 30-day aortic event outcomes of patients with acute uncomplicated type B IMH. We identified ULP, MDAD, anemia, and absence of diabetes as independent predictors of short-term aortic-related adverse events in patients with type B IMH, based on clinical characteristics and CTA. The nomogram based on clinical and imaging features can be used to predict the short-term prognosis of type B IMH patients treated with OMT, and it can provide valuable assistance in clinical decision-making.

## Data Availability

The raw data supporting the conclusions of this article will be made available by the authors, without undue reservation.

## References

[1] Isselbacher EM Preventza O Hamilton Black J Augoustides JG Beck AW Bolen MA 2022 ACC/AHA guideline for the diagnosis and management of aortic disease: a report of the American heart association/American college of cardiology joint committee on clinical practice guidelines. Circulation. (2022) 146:e334–482. 10.1161/CIR.0000000000001106 36322642 PMC9876736

[2] Evangelista A Mukherjee D Mehta RH O'Gara PT Fattori R Cooper JV Acute intramural hematoma of the aorta: a mystery in evolution. Circulation. (2005) 111:1063–70. 10.1161/01.CIR.0000156444.26393.80 15710757

[3] von Kodolitsch Y Csösz SK Koschyk DH Schalwat I Loose R Karck M Intramural hematoma of the aorta: predictors of progression to dissection and rupture. Circulation. (2003) 107:1158–63. 10.1161/01.cir.0000052628.77047.ea 12615795

[4] Erbel R Aboyans V Boileau C Bossone E Bartolomeo RD Eggebrecht H 2014 ESC guidelines on the diagnosis and treatment of aortic diseases: document covering acute and chronic aortic diseases of the thoracic and abdominal aorta of the adult. The task force for the diagnosis and treatment of aortic diseases of the European Society of Cardiology (ESC). Eur Heart J. (2014) 35:2873–926. 10.1093/eurheartj/ehu281 25173340

[5] Li Z Lu B Chen Y Hou Z Chen B Zhang Y Acute type B aortic intramural hematoma: the added prognostic value of a follow-up CT. Eur Radiol. (2019) 29:6571–80. 10.1007/s00330-019-06254-0 31144073

[6] Mesar T Lin MJ Kabir I Hou Z Chen B Zhang Y Medical therapy in type B aortic intramural hematoma is associated with a high failure rate. J Vasc Surg. (2020) 71:1088–96. 10.1016/j.jvs.2019.07.084 32063446

[7] Liu YJ Zhang QY Du ZK Yang L Zhang L He RX Long-term follow-up and clinical implications in Chinese patients with aortic intramural hematomas. Int J Cardiol. (2018) 270:268–72. 10.1016/j.ijcard.2018.06.077 29945807

[8] Sailer AM Nelemans PJ Hastie TJ Chin AS Huininga M Chiu P Prognostic significance of early aortic remodeling in acute uncomplicated type B aortic dissection and intramural hematoma. J Thorac Cardiovasc Surg. (2017) 154:1192–200. 10.1016/j.jtcvs.2017.04.064 28668458 PMC5603396

[9] Mussa FF Horton JD Moridzadeh R Nicholson J Trimarchi S Eagle KA. Acute aortic dissection and intramural hematoma: a systematic review. JAMA. (2016) 316:754–63. 10.1001/jama.2016.10026 27533160

[10] Evangelista A Maldonado G Moral S Teixido-Tura G Lopez A Cuellar H Intramural hematoma and penetrating ulcer in the descending aorta: differences and similarities. Ann Cardiothorac Surg. (2019) 8:456–70. 10.21037/acs.2019.07.05 31463208 PMC6687957

[11] Ishizu K Kaji S Nakashima M Kitai T Kim K Ehara N Focal intimal disruption size at multidetector CT and disease progression in type B aortic intramural hematoma. Radiology. (2021) 301:311–9. 10.1148/radiol.2021204385 34374587

[12] Piazza M Squizzato F Porcellato L Casali E Grego F Antonello M. Predictors of intervention in acute type B aortic penetrating ulcer and intramural hematoma. Semin Thorac Cardiovasc Surg. (2024) 36:1–10. 10.1053/j.semtcvs.2022.07.009 35931348

[13] Moral S Cuéllar H Avegliano G Ballesteros E Salcedo MT Ferreira-González I Clinical implications of focal intimal disruption in patients with type B intramural hematoma. J Am Coll Cardiol. (2017) 69:28–39. 10.1016/j.jacc.2016.10.045 28057247

[14] Alomari IB Hamirani YS Madera G Tabe C Akhtar N Raizada V. Aortic intramural hematoma and its complications. Circulation. (2014) 129:711–6. 10.1161/CIRCULATIONAHA.113.001809 24515957

[15] Kitai T Kaji S Yamamuro A Tani T Kinoshita M Ehara N Detection of intimal defect by 64-row multidetector computed tomography in patients with acute aortic intramural hematoma. Circulation. (2011) 124:S174–8. 10.1161/CIRCULATIONAHA.111.037416 21911809

[16] Wei C Li J Du E Miao Y Li P Guan W. Clinical and imaging differences between Stanford type B intramural hematoma-like lesions and classic aortic dissection. BMC Cardiovasc Disord. (2023) 23:378. 10.1186/s12872-023-03413-6 37507680 PMC10386763

[17] Wu MT Wang YC Huang YL Chang RS Li SC Yang P Intramural blood pools accompanying aortic intramural hematoma: CT appearance and natural course. Radiology. (2011) 258:705–13. 10.1148/radiol.10101270 21212368

[18] Park GM Ahn JM Kim DH Kang JW Song JM Kang DH Distal aortic intramural hematoma: clinical importance of focal contrast enhancement on CT images. Radiology. (2011) 259:100–8. 10.1148/radiol.11101557 21330562

[19] Chao CP Walker TG Kalva SP. Natural history and CT appearances of aortic intramural hematoma. Radiographics. (2009) 29:791–804. 10.1148/rg.293085122 19448116

[20] Shao T Bornak A Kang N. Penetrating aortic ulcer and aortic intramural hematoma: treatment strategy. Vascular. (2023) 31(6):1086–93. 10.1177/17085381221102785 35578772

[21] Li Z Liu C Wu R Zhang J Pan H Tan J Prognostic value of clinical and morphologic findings in patients with type B aortic intramural hematoma. J Cardiothorac Surg. (2020) 15:49. 10.1186/s13019-020-1067-8 32293486 PMC7092490

[22] Kruse MJ Johnson PT Fishman EK Zimmerman SL. Aortic intramural hematoma: review of high-risk imaging features. J Cardiovasc Comput Tomogr. (2013) 7:267–72. 10.1016/j.jcct.2013.04.001 23770125

[23] Gifre-Renom L Jones EAV. Vessel enlargement in development and pathophysiology. Front Physiol. (2021) 12:639645. 10.3389/fphys.2021.639645 33716786 PMC7947306

[24] Shen ZJ He XW. A case-control study on the relationship between diabetes and the risk of aortic intramural hematoma. Chinese J of Evid Based Cardiovasc Med. (2018) 10:1537–9.

[25] Takagi H Umemoto T. Negative association of diabetes with thoracic aortic dissection and aneurysm. Angiology. (2017) 68:216–24. 10.1177/0003319716647626 27166380

[26] Tsai CL Lin CL Wu YY Shieh DC Sung FC Kao CH. Advanced complicated diabetes mellitus is associated with a reduced risk of thoracic and abdominal aortic aneurysm rupture: a population-based cohort study. Diabetes Metab Res Rev. (2015) 31:190–7. 10.1002/dmrr.2585 25066630

[27] He X Liu X Liu W Wang B Liu Y Li Z Association between diabetes and risk of aortic dissection: a case-control study in a Chinese population. PLoS One. (2015) 10:e0142697. 10.1371/journal.pone.0142697 26562793 PMC4643043

[28] Chen Q Jiang D Kuang F Yang F Shan Z. Outcomes of uncomplicated type B intramural hematoma patients with type 2 diabetes mellitus. J Card Surg. (2021) 36:1209–18. 10.1111/jocs.15336 33462880

[29] Golledge J Karan M Moran CS Muller J Clancy P Dear AE Reduced expansion rate of abdominal aortic aneurysms in patients with diabetes may be related to aberrant monocyte-matrix interactions. Eur Heart J. (2008) 29:665–72. 10.1093/eurheartj/ehm557 18263873

[30] Dua MM Miyama N Azuma J chultz GM Sho M Morser J Hyperglycemia modulates plasminogen activator inhibitor-1 expression and aortic diameter in experimental aortic aneurysm disease. Surgery. (2010) 148:429–35. 10.1016/j.surg.2010.05.014 20561659 PMC2905480

[31] Prakash SK Pedroza C Khalil YA Milewicz DM. Diabetes and reduced risk for thoracic aortic aneurysms and dissections: a nationwide case-control study. J Am Heart Assoc. (2012) 1:1–8. 10.1161/JAHA.111.000323 23130125 PMC3487378

